# Dynamic Simulations on the Arachidonic Acid Metabolic Network

**DOI:** 10.1371/journal.pcbi.0030055

**Published:** 2007-03-23

**Authors:** Kun Yang, Wenzhe Ma, Huanhuan Liang, Qi Ouyang, Chao Tang, Luhua Lai

**Affiliations:** 1 Beijing National Laboratory for Molecular Sciences, State Key Laboratory for Structural Chemistry of Unstable and Stable Species, College of Chemistry and Molecular Engineering, Peking University, Beijing, China; 2 Center for Theoretical Biology, Peking University, Beijing, China; European Molecular Biology Laboratory, Germany

## Abstract

Drug molecules not only interact with specific targets, but also alter the state and function of the associated biological network. How to design drugs and evaluate their functions at the systems level becomes a key issue in highly efficient and low–side-effect drug design. The arachidonic acid metabolic network is the network that produces inflammatory mediators, in which several enzymes, including cyclooxygenase-2 (COX-2), have been used as targets for anti-inflammatory drugs. However, neither the century-old nonsteriodal anti-inflammatory drugs nor the recently revocatory Vioxx have provided completely successful anti-inflammatory treatment. To gain more insights into the anti-inflammatory drug design, the authors have studied the dynamic properties of arachidonic acid (AA) metabolic network in human polymorphous leukocytes. Metabolic flux, exogenous AA effects, and drug efficacy have been analyzed using ordinary differential equations. The flux balance in the AA network was found to be important for efficient and safe drug design. When only the 5-lipoxygenase (5-LOX) inhibitor was used, the flux of the COX-2 pathway was increased significantly, showing that a single functional inhibitor cannot effectively control the production of inflammatory mediators. When both COX-2 and 5-LOX were blocked, the production of inflammatory mediators could be completely shut off. The authors have also investigated the differences between a dual-functional COX-2 and 5-LOX inhibitor and a mixture of these two types of inhibitors. Their work provides an example for the integration of systems biology and drug discovery.

## Author Summary

Inflammation is a basic way in which the body reacts to infection, irritation, or other injury. When it is uncontrolled and misdirected, it causes diseases such as rheumatoid arthritis, inflammatory bowel disease, asthma, and others. In the United States, more than 1% of the population uses nonsteroidal anti-inflammatory drugs, such as aspirin, ibuprofen, or naproxen, daily to relieve aches and pains. However, these drugs have undesirable side effects. The withdrawal of VIOXX (rofecoxib; Merck, http://www.merck.com) in 2004 has given a good lesson on safety problems. To assist the design of safe anti-inflammatory drugs, we have constructed a computational model of the arachidonic acid (AA) metabolic network in human polymorphous leukocytes. By analyzing the flux changes upon drug treatment in this metabolic network, drugs against multiple targets were found to be capable of reducing toxicity as they exhibited balanced control of the system. The model of the AA metabolic network provides helpful information for anti-inflammatory drug discovery. This work sets an example for the integration of systems biology and drug discovery.

## Introduction

Nonsteriodal anti-inflammatory drugs (NSAIDs) (e.g., aspirin) are widely used for the treatment of musculoskeletal pain and other conditions. In the US, more than 1% of the population uses NSAIDs daily [1], and the market for NSAIDs now amounts to more than $6 billion annually worldwide [2]. Although NSAIDs do alleviate the aches and pains, these drugs have undesirable side effects on the gastrointestinal tract and the central nervous system in addition to the potential exacerbation of conditions such as asthma [1]. The findings that cyclooxygenase-2 (COX-2) plays a major role in inflammation, and that inhibition of COX-1 causes gastrointestinal toxicity and mild bleeding diathesis [3], had suggested that selective COX-2 inhibitor would be an effective anti-inflammatory drug with low gastrointestinal side effects [4]. Ironically, the unexpected cardiovascular side effects of selective COX-2 inhibitors have surfaced [5,6]. Thus, on September 30, 2004, Merck & Company announced a voluntary withdrawal of the company's COX-2 inhibitor, VIOXX (rofecoxib) [7]. Other FDA-approved COX-2 inhibitors, such as celecoxib (Celebrex) and valdecoxib (Bextra), are being re-evaluated [8–10]. Despite years of studies, safe anti-inflammatory drug design remains a great challenge.

Failures in anti-inflammatory drug design illustrate the limitations of the current drug discovery paradigm. A steady waning in the productivity of the pharmaceutical industry in the past decade has been observed. This decline coincides with the introduction of target-based drug discovery [11]. Recently, medicinal chemists have started to think about drug discovery from a systems biology perspective [12,13]. Studying the cross-talks between biological responses rather than one by one may provide a better understanding of disease development and achieve accurate evaluation on drug efficacy and toxicity [14,15]. This new approach has been applied to safe drug design [16,17]. For example, the former SmithKline Beecham (now GlaxoSmithKline, http://www.gsk.com) focused on the blood coagulation cascade biochemical network [18,19]. Armed with a good understanding of the disease from the regulatory network level, the company used model predictions to develop a fully humanized anti–Factor IX antibody that has entered clinical trials. Rajasethupathy et al. have recently reviewed advances in the practical applications of systems biology to drug discovery [20]. These researchers promote the development of network-based drug design, which devises drug-treatment strategies from the level of the disease system using computational models and high-throughput experiments.

In this paper, we study the dynamic properties of the arachidonic acid (AA) metabolic network in human polymorphonuclear leukocytes (PMNs) in the hope of gaining more insights into anti-inflammatory drug design. An ordinary differential equation (ODE) model of the AA metabolic network was developed. Flux analysis and simulation on the addition of exogenous AA were performed to study the network balance. The therapeutic effects of anti-inflammatory inhibitors were simulated, and the difference between dual functional COX-2 and 5-lipoxygenase (5-LOX) inhibitors and the mixture of these two types of inhibitors was studied. Corresponding experiments on the introduction of exogenous AA, COX-2, and 5-LOX inhibitors were performed and were found to be consistent with model predictions. Our work shows that flux balance is important for the efficacy and safety of the drugs. Compared with traditional single-target drugs, drugs against multiple targets can control the network balance and lead to safer treatment.

## Results

### The Metabolic Network of AA in Human PMNs

**Figure 1:**
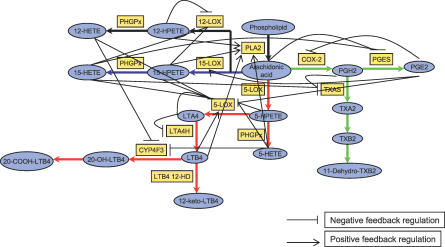
The AA Metabolic Network in Human PMNs AA is metabolized through three main pathways: the 5-LOX pathway (red line), the 15-LOX pathway (blue line), and the COX-2 pathway (green line). PGE2, LTA4, and LTB4 are the major inflammatory mediators produced in the COX-2 and 5-LOX pathways. A total of 24 feedback loops, which are involved in the network, make important contributions to the regulation of inflammatory mediators.

Inflammation is a type of nonspecific immune response to infection, irritation, or other injury. It is characterized by redness, swelling, pain, and sometimes loss of function. When harmful agents invade the human body, inflammatory mediators are released by immune cells. This release causes vasodilation, emigration of neutrophils, chemotaxis, and increased vascular permeability. AA is the precursor of inflammatory mediators, including prostaglandins (PGs) and leukotrienes (LTs). Extensive researches on the metabolism of AA in human PMNs have been performed. Thus, based on the Kyoto Encyclopedia of Genes and Genomes (KEGG) [21] and a survey of the literature (Figure 1; see details in Protocol S1), a computational model of the AA metabolic network in human PMNs was constructed (Dataset S1).

**Figure 2:**
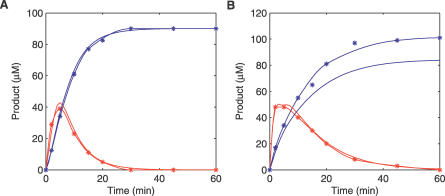
Comparison of the Experimental Data and Computational Results PMNs (20 × 10^6^ cells/ml) were incubated at 37 °C with 10 μM A23187 (a calcium ionophore) (A) and 10 μM A23187 + 30 μM AA (B), respectively. The calculated results (line without star: red for LTB4 and blue for ω-LTB4) fit well to experimental data [22] (line with star: red for LTB4 and blue for ω-LTB4), considering experimental error.

The production of inflammatory mediators is initiated by inflammatory stimuli that activate phospholipase A2. This enzyme catalyzes the hydrolysis of the sn-2 position of the membrane glycerophospholipids to release AA. Two separate pathways produce inflammatory mediators. One is initiated by COX-2 and produces PGs, while the other is initiated by 5-LOX and produces LTs. PGE2, LTA4, and LTB4 are the major inflammatory mediators in our model. Once PMNs have been activated by inflammatory stimuli, the cells release inflammatory mediators and cause accumulation and activation of other cells. A series of ODEs was established to simulate unicellular behavior (see details in Materials and Methods), which included 24 initial concentrations and 45 reaction constants (see details in Protocol S1). A total of 23 reaction constants was taken from experimental values, while the others were obtained by fitting the calculated production of LTB4 and its ω-oxidation products (ω-LTB4) to experimental data [22] (Figure 2 and Tables S1–S2). The parameter set that fit the experimental data well was chosen for further studies.

### Dynamic Properties of the Network

**Figure 3:**
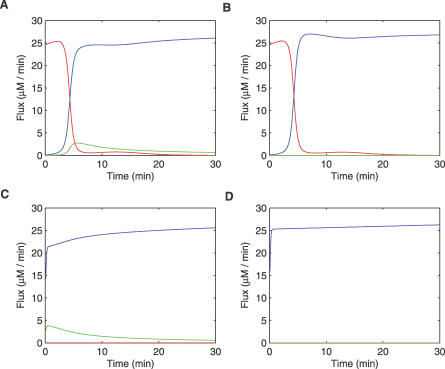
Flux Analysis on the Three Main Pathways: The 5-LOX Pathway, the 15-LOX Pathway, and the COX-2 Pathway Four states were studied: the disease state with no addition of inhibitors (A), the inhibited state with addition of COX-2 inhibitor (B), addition of 5-LOX inhibitor (C), or addition of both COX-2 and 5-LOX inhibitors (D). Red line, 5-LOX pathway; blue line, 15-LOX pathway; and green line, COX-2 pathway.

Flux analysis was performed on the main network pathways, which included the 5-LOX, 15-LOX, and COX-2 pathways. The result of the 12-LOX pathway analysis was not shown here due to its weak effect on other pathways in the current network [23]. Figure 3A shows simulations of 30 min. In the first 5 min, following the activation by inflammatory stimuli, the flux of the 5-LOX pathway was the largest and was responsible for the major metabolism of AA. As the result of negative feedback regulations (e.g., the suicide inhibition of LTA4H by LTA4), the flux of the 5-LOX pathway decreased along with time, while the flux of the 15-LOX pathway increased. Ultimately, the 15-LOX pathway flux became the largest after the first 5 min. During the 30-min simulation, the flux of the COX-2 pathway remained small and played the least important role in the AA metabolic network in human PMNs. The first 10 min was critical for the production of LTs, while PGs continued to accumulate with time. These findings are consistent with previous experimental data [24,25] that showed that LTs rather than PGs are the main inflammatory mediators produced in human PMNs.

**Figure 4:**
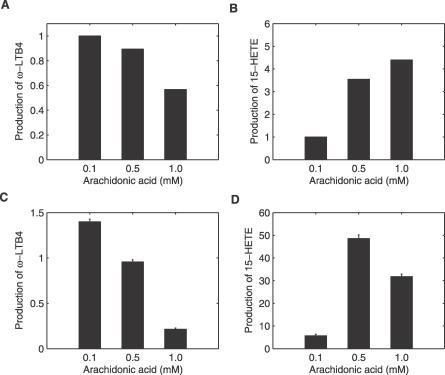
Effects of Exogenous AA on the Network Balance The 1-h output of ω-LTB4 (20-OH-LTB4 + 20-COOH-LTB4) (A) and 15-HETE (B) were calculated to evaluate changes in the 5-LOX pathway and the 15-LOX pathway. To validate the simulation, experiments were performed: PMNs (1.66 × 10^7^ cells/ml) were incubated at 37 °C with 10 μM A23187 (a calcium ionophore) and arachidonic acid of different concentration for 1h. The output of ω-LTB4 (C) and 15-HETE (D) were measured. The *y*-axis represents the production with exogenous AA normalized against that without exogenous AA.

Further simulations with different exogenous AA concentrations were performed. It seems logical to postulate that more AA would produce more inflammatory mediators because AA was the only source of LTs and PGs in our model; however, the simulation results interestingly showed that the output of LTs decreased with the concomitant increase of exogenous AA (Figure 4A and 4D). This decrease was the result of the negative-feedback mechanisms in the 5-LOX pathway. The additional AA is mainly metabolized through the 15-LOX pathway. Further experiments were performed to validate this assumption (see details in Materials and Methods). A decrease of LTs was observed when more than 0.5 mM AA was added to PMNs. The output of (15*S*)-hydroxy-5*Z*,8*Z*,11*Z*,13*E*-eicosatetraenoic acid (15-HETE) increased at the same time (Figure 4C and 4B). Thus, the simulation predictions are qualitatively consistent with the experimental results.

### Simulating the Therapeutic Effects of Anti-Inflammatory Inhibitors

**Figure 5:**
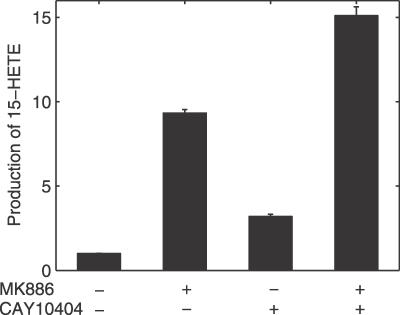
The Production of 15-HETE in the Presence of Inhibitors PMNs (1.66 × 10^7^ cells/ml) were preincubated with inhibitors for 10 min at 37 °C and then were stimulated with 10 μM A23187 (a calcium ionophore) for 1 h at 37 °C. MK886, an inhibitor of 5-LOX, was used to block the production of LTs. CAY10404, an inhibitor of COX-2, was used to block the production of PGs. The *y*-axis represents the production of 15-HETE with exogenous AA normalized against that without exogenous AA.

Simulations on widely used anti-inflammatory inhibitors, including 5-LOX inhibitor, COX-2 inhibitor, and a combination of the two, were performed (Figure 3). The COX-2 inhibitor increased the flux through the 15-LOX pathway, while the flux through the 5-LOX pathway remained almost constant. The reason is that the effect of COX-2 pathway in the current model is weak in the early stage of AA metabolism and becomes more evident with time. The 5-LOX inhibitor induced the peak flux immediately after initiating the metabolism in both the COX-2 and the 15-LOX pathways. When both the COX-2 and 5-LOX inhibitors were introduced, the COX-2 and 5-LOX pathways were shut off, and the majority of the flux went through the 15-LOX pathway. These simulation results showed that single-target anti-inflammatory drugs cannot stop the production of all inflammatory mediators, and that the combination of the 5-LOX and COX-2 inhibitors would likely yield better therapeutic results. All of the inhibitors increased the production of 15-HETE, which was consistent with our experimental results (Figure 5).

We then did further simulation on the combination of 5-LOX inhibitor and COX inhibitor. There are two strategies for this combination: developing dual functional COX-2 and 5-LOX inhibitors, or using a mixture of these two types of inhibitors. A few papers have been published on the development of these two anti-inflammatory strategies, and some drug candidates have already been in clinical tests [26–31]. When using a combination of inhibitors, two issues need to be considered: one is the mixing ratio (MR) of different single-functional inhibitors, which makes great contributions to the efficacy and safety of the mixture; the other is the relative inhibition constant to different enzymes (DR), which decides the therapeutic effect of the dual-functional inhibitor. The efficacy of the dual-functional COX-2 and 5-LOX inhibitor and the mixture with different concentrations and DR/MR values was investigated and compared. To ensure the equality in the comparison, the same total inhibition ability (Ki_COX-2_ × Ki_5-LOX_; see details in Materials and Methods) and the same total concentration of inhibitors were used in the simulations. The inhibition intensity on the production of LTs and PGs was calculated to evaluate the efficacy of inhibitors (see details in Materials and Methods).

**Figure 6:**
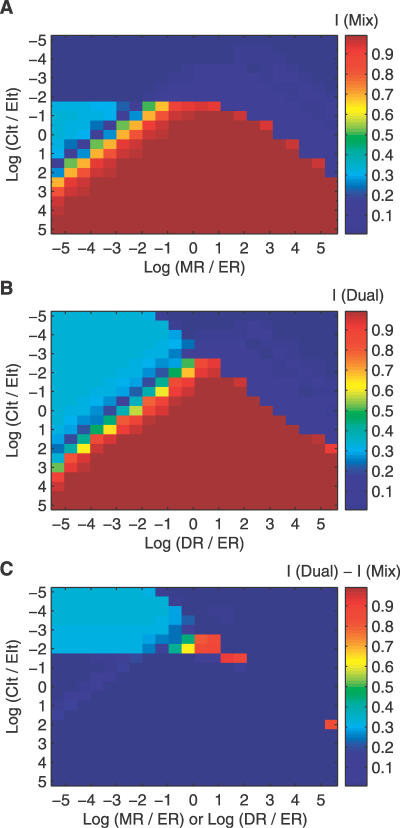
The Effect of the Mixture and the Dual-Functional COX-2/5-LOX Inhibitors (A) The effect of MR on the efficacy of the mixture of COX-2/5-LOX inhibitors was simulated. (B) The effect of DR on the efficacy of the dual functional COX-2/5-LOX inhibitor was simulated. (C) Comparison of the therapeutic effects of these two kinds of inhibitors. The inhibition intensity on the production of LTs and PGs was calculated to evaluate the efficacy of inhibitors, where I (Mix) is the inhibition intensity of the mixture and I (Dual) is the inhibition intensity of the dual functional inhibitor. ER is the relative activity of COX-2 to 5-LOX, which is 0.02 in the current model. CIt is the total concentration of inhibitors while CEt is the total concentration of COX-2 and 5-LOX. DR is the relative inhibition constant to different enzymes (DR = *Ki*_5-LOX_ / *Ki*_COX-2_), and MR is the mixing ratio of the COX-2 inhibitor and the 5-LOX inhibitor (MR = [COX-2] / [5-LOX]). The efficacy of these two types of inhibitors was compared by the value of difference between I(Dual) and I(Mix).

As shown in Figure 6A, for the mixture, the inhibitors had the largest effective concentration region when MR was close to the relative activity (ER) of the two enzymes (see definition in Materials and Methods); that is, the enzymes can be inhibited to more than 90% if the total inhibitor concentration (CIt) was larger than approximately 1% of the total concentration of 5-LOX and COX-2 (CEt). When CIt was less than 1% of CEt, the enzymes cannot be effectively inhibited. When MR deviated from ER, the effective concentration region of the inhibitors became much smaller (e.g., when MR deviated from ER by an order of 10^4^), strong inhibition (>90%) was achieved only when CIt was about ten times larger than the CEt. Davies et al. [32] have reviewed the clinical pharmacokinetics of a COX-2–selective inhibitor, meloxicam. The maximum plasma concentration of this compound is from 0.531 to 5.35 mg/l after application of clinical dosage (5–30 mg) to volunteers, which is about 0.3 to 3 times the CEt in our model. Thus, in general clinical treatments, the mixture of compounds might be effective in a quite broad region of MR/ER (about 0.001 to 1,000). If MR deviates too much from ER, then the clinical dosage will not be effective.

For the dual-functional inhibitor, the compound also had the largest effective concentration region when DR was close to ER (Figure 6B). However, the low concentration limit was extended to as low as 10^−4^ (CIt/CEt); that is, the dual-functional inhibitor was much more effective compared with the mixture of inhibitors when the concentration was low (Figure 6C). If the dual-functional inhibitors and the mixture have similar pharmacokinetic properties, and the same drug dose is applied to patients, the dual-functional inhibitors will be effective for a longer time than the mixture, as they are active at low concentration. When DR deviated from ER, the effective concentration region of the inhibitor also became smaller. There was a similar trend when DR was larger than ER. When DR was smaller than ER, noticeable inhibition could be observed at very low inhibitor concentration.

## Discussion

Development of safe anti-inflammatory drugs has been an especially tough problem for a long time. Failures in selective COX-2 inhibitors have provided a good lesson on the drug safety problem. Thus, network-based drug design, following the development of systems biology, should be useful for finding safe therapeutic strategies from the level of the disease network. In the current study, we used the AA metabolic network in human PMNs as an example for the network-based drug design study. A mathematical model for the AA metabolic network in human PMNs was built using ODEs to describe enzymatic reactions and feedback loops. Some of the parameters were taken from published experimental studies, and other parameters and initial conditions were determined by fitting to experimental curves of LTB4 and derivative production. The model was validated by experiments on the influence of introducing exogenous AA or inhibitors and was found to explain experimental data well. However, as the network is complex, its robustness and reliability need to be further validated in future studies. Nevertheless, our AA metabolic network model can enhance the understanding of the production time course of inflammatory mediators, including LTs and PGs, and can be used to assist anti-inflammatory drug design.

Network dynamic properties were found to be important for anti-inflammatory drug design targeting the AA network. Results of flux analysis, simulations, and experiments on the effect of exogenous AA showed the complexity of a compound's behavior in a system. Due to the effect of feedback regulations and other pathways in the network, the effect of a molecule in a system may not be same as its effect on a single point reaction. Thus, designing drugs from the network system level is necessary. Performing model studies combined with experiments would be an effective way to find safe drugs.

Single-target anti-inflammatory inhibitors were found to have limitations based on simulations and experiments in the current work. Single-target inhibitors cannot achieve full success in anti-inflammatory treatment because they cannot control the production of both LTs and PGs at the same time. Results in our studies suggest that the combination of the COX-2 inhibitor and the 5-LOX inhibitor would have better treatment. In fact, a dual COX-2/5-LOX inhibitor, licofelone (ML3000) [33,34], has been successfully completed phase III trials and is demonstrated to be superior in safety and equally efficacious for standard treatments of osteoarthritis.

Furthermore, multitarget anti-inflammatory drug efficacy was investigated. We simulated and compared the efficacy of dual COX-2/5-LOX inhibitors and the mixture of single-functional COX-2 and 5-LOX inhibitors. The mixing ratio and the relative inhibition constant were found to be important to the efficacy of the mixture and the dual-functional inhibitor, respectively. Generally speaking, the dual-functional inhibitor was effective in a larger concentration range, making it more robust towards concentration fluctuations. Further studies are necessary to achieve a better understanding of the difference between the dual-functional inhibitor and the mixture of single-functional inhibitors.

## Materials and Methods

### Simulation methods.

*The mathematical model of the AA metabolic network in PMNs.* Based on KEGG and a survey of the literature, a group of ODEs were devised to develop the mathematical model of the AA metabolic network in human PMNs (see details in Protocol S1 and Dataset S1). Michaelis–Menten equations are used to describe the catalysis in the network:

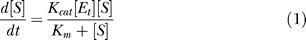
where [*S*] is the concentration of substrate, [*E_t_*] is the total concentration of enzyme, *K_cat_* is turnover number, and *K_m_* is the Michaelis–Menten constant.

If competitive reversible inhibitors are involved in the catalysis, the equation is:

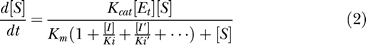
where [*I*] is the concentration of inhibitor and *Ki* is the inhibition constant, which is defined as:

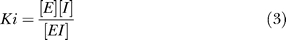



If the inhibitors are irreversible, we assume the enzymes would decay according to the following equation:

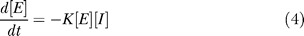
where *K* is a constant.

When activators are involved in the catalysis, we use the following equation:

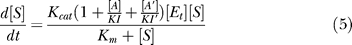
where [*A*] is the concentration of activator and *KI* is a constant.

PGE2 can upregulate 15-LOX through transcription in this network. Based on the experimental data, we describe its effect with the following equation:

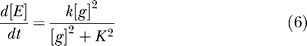
where [*g*] is the concentration of PGE2 and *K* is a constant.

The ode15s routine of Matlab 6.5 (Mathworks, http://www.mathworks.com) was used to solve the ODEs. LTB4 and ω-LTB4 metabolic curves under different exogenous AA concentrations were calculated. These calculated curves were fit to the experimental data by empirically modulating parameters that had no direct values from published experiments, while the other parameters remained fixed to their experimental values. The parameter set that fit the experimental data well was chosen for further studies.

*Simulating the therapeutic effects of the dual functional inhibitor and the mixture.* The inhibition behavior on different enzymes is assumed to be independent and can be calculated by the following equations (only competitive reversible inhibitors are studied here):

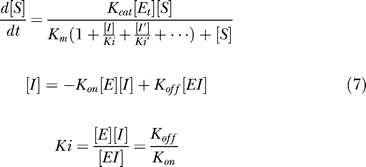
where [*I*] is the concentration of inhibitor, and *Ki* is the dissociation constant. We use these equations with the consideration that the binding affinity of drugs is usually strong and the necessary concentration of inhibitors is in the same magnitude with the concentration of the enzyme. Then the enzyme-inhibitor complex cannot be neglected, and the above equations are required.

To evaluate the efficacy of inhibitors, inhibition intensity on the production of inflammatory mediators is defined as:

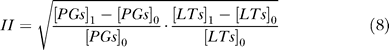
where [*PGs*]_1_ is the concentration of PGs after taking drugs, [*PGs*]_0_ is the concentration of PGs before treatment, [*LTs*]_1_ is the concentration of LTs after taking drugs, and [*LTs*]_0_ is the concentration of LTs before treatment.

We use the same value of total inhibition ability and total concentration of the dual functional inhibitor and the mixture to ensure the equality in the comparison. The total inhibition ability is defined as the product of inhibit constant to COX-2 and 5-LOX (Ki_COX-2_ × Ki_5-LOX_) and is fixed to 1 × 10^−14^ in all simulations. ER is defined as:

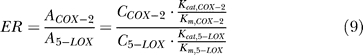
where *A* is the activity of enzyme, and *C* is the concentration. The ER value in the current model is 0.02.

### Experiments.

*Materials.* LTB4, 20-OH-LTB4, 20-COOH-LTB4, 5-HETE, 15-HETE, PGB2, MK886, CAY10404, and AA were purchased from Cayman Chemical (http://www.caymanchem.com). The calcium ionophore, A23187, was obtained from Acros Organics (http://www.acros.com). Concentrations of the LTs and hydroxyeicosatetraenoic acids were determined by UV spectroscopy at 270 nm and 236 nm, respectively. PGB2 was used as an internal standard, and its concentration was determined by UV spectroscopy at 278 nm [ɛ(PGB2) = 26,000].

### Preparation of PMNs from human blood.

Following the reported procedure [35], human PMNs were isolated from the venous blood of healthy volunteers who had not ingested any aspirin-like compounds in the preceding week. Blood was collected in the anticoagulant Na-EDTA and was centrifuged at 100*g* for 10 min. The platelet-rich plasma was discarded. The blood was then mixed with 6% dextran T-500 in 0.01 M PBS (T500/PBS = 1:1). After 45 min, the upper layer that contained the leukocytes was layered gently over lymphocyte separation medium and centrifuged at 550*g* for 30 min to pellet the cells. The residual erythrocytes were removed by osmotic hemolysis with ice-cold water for 20 s. The mixture was washed twice by centrifugation in 0.01 M PBS at 250*g* for 5 min and then resuspended in PBS (>95% PMNs, <5% monocytes and lymphocytes).

### Effect of inhibitors on the network.

PMNs were preincubated with MK886 [36] (an inhibitor of 5-LOX), CAY10404 [37] (an inhibitor of COX-2), and a combination of MK886 and CAY10404 at 37 °C for 10 min, respectively. To stimulate the PMNs, 10 μM A23187, 2 mM Ca^2+^, and 2 mM  Mg^2+^ were added for 1 h at 37 °C. Incubations were terminated by the addition of ethyl acetate (containing 160 μl acetic acid/40 ml). PGB2 (100 ng) was added as an internal standard. The upper solvent was evaporated under a stream of nitrogen, and the residue was dissolved in 30 μl methanol.

Reverse-phase high-performance liquid chromatography (HPLC) was used to assay 20-OH-LTB4, 20-COOH-LTB4, and 15-HETE [38]. HPLC was performed with an Agilent 1100 series instrument (http://www.agilent.com). A column (Retasil C18, 4.6 × 200 mm cartridge, 5 μm particle size; Elite, http://las.perkinelmer.com) was used for separation of the samples. Solvents A and B consisted of methanol–water–acetic acid (10:90:0.05) and methanol–acetonitrile–acetic acid (30:70:0.05), respectively. Lipids were eluted at a rate of 1.0 ml/min with continuous monitoring for UV absorbance at 235 nm and 270 nm for detection of 15-HETE and LTs, respectively. The retention times for 20-COOH-LTB4, 20-OH-LTB4, PGB2, and 15-HETE were 6.399, 6.603, 11.604, and 17.899 min, respectively.

### Effect of AA on the network.

Cells were preincubated with 0.1 mM, 0.5 mM, and 1 mM AA at 37 °C for 10 min. To stimulate PMNs, 10 μM A23187, 2 mM Ca^2+^, and 2 mM Mg^2+^ were then added for 1 h at 37 °C. Incubations were terminated by the addition of ethyl acetate (containing 160 μl acetic acid/40 ml). PGB2 (100 ng) was added as an internal standard. The upper solvent was evaporated under a stream of nitrogen, and the residue was dissolved in 30 μl methanol. Again, reverse-phase HPLC was used to assay 20-OH-LTB4, 20-COOH-LTB4, and 15-HETE as described above.

## Supporting Information

Dataset S1 The SBML Model of the AA Metabolic Network in Human PMNs The serial number of metabolites, enzymes, and feedback regulations used in the model is defined in Protocol S1. Parameters are summarized in Tables S1 and S2.
(67 KB XML)

Protocol S1 Details of the AA Metabolic Network Model That Are Not Mentioned in the Main Text The AA metabolic network in human PMNs is constructed based on KEGG and a survey of the literature. References used for model construction and 24 ODEs used in simulations are listed.
(214 KB DOC)

Table S1 Parameters of Feedback Regulations Used in the Model (55 KB DOC)

Table S2 The *K_m_* and *K_cat_* of Enzymes Used in the Model (34 KB DOC)

## Abbreviations

5-LOX: 5-lipoxygenase
AA: arachidonic acid
COX: cyclooxygenase
DR: relative inhibition constant to different enzymes
ER: relative activity of enzymes
KEGG: Kyoto Encyclopedia of Genes and Genomes
LT: leukotriene
MR: mixing ratio
NSAID: nonsteriodal anti-inflammatory drug
ODE: ordinary differential equation
PG: prostaglandin
PMN: polymorphonuclear leukocyte
