# Supplementary material for: Dynamic Simulations on the Arachidonic Acid Metabolic Network
Source: PLoS Comput Biol. 2007 Mar 23;3(3):e55. doi: 10.1371/journal.pcbi.0030055 (PMC1829479; doi:10.1371/journal.pcbi.0030055)
Supplement: Supplementary file 1 [file pcbi.0030055.sd002.doc]

**Dynamic simulations on the Arachidonic Acid Metabolic Network**

Kun Yang^1,2^, Wenzhe Ma^1,2^, Huanhuan Liang^1^, Qi Ouyang^2^, Chao Tang^2^, Luhua Lai^1,2*^

^1^Beijing National Laboratory for Molecular Sciences, State Key Laboratory for Structural Chemistry of Unstable and Stable Species, College of Chemistry and Molecular Engineering, Peking University, Beijing 100871, China.

^2^Center for Theoretical Biology, Peking University, Beijing 100871, China

**Protocol S1**

The arachidonic acid (AA) metabolic network in human PMNs was constructed based on the published experimental data[1-22] (Fig.S1). A mathematical model has been set up to simulate the dynamic properties of the network and assist the anti-inflammatory drug design approaches. The calculation curves of LTB4 and ω-LTB4 production were fitted to the experimental data. The ordinary differential equations used in the model are shown below. The serial number of products, enzymes and feedback regulations is defined in Figure S1 and the values of dynamic parameters used in the simulation are listed in Table S1 and Table S2.

$\frac{d[AA]}{dt}=\frac{K15(1+\frac{[12-HPETE]}{KI19}+\frac{[15-HPETE]}{KI20}+\frac{[LTB4]}{KI21}+\frac{[5-HETE]}{KI22})[PLA2][PS]}{k15(1+\frac{[AA]}{Ki})+[PS]}$ $-\frac{K16[15-LOX][AA]}{k16(1+\frac{[15-HPETE]}{Ki})+[AA]}-\frac{K17[12-LOX][AA]}{k17(1+\frac{[12-HPETE]}{Ki18}+\frac{[15-HETE]}{Ki16})+[AA]}$ $-\frac{K18[COX][AA]}{k18(1+\frac{[PGH2]}{Ki}+\frac{[PGE2]}{Ki3})+[AA]}$

$$-\frac{K21[5-LOX][AA]}{k21(1+\frac{[5-HPETE]}{Ki}+\frac{[12-HETE]}{Ki7}+\frac{[15-HETE]}{Ki8}+\frac{[PGE2]}{Ki11}+\frac{[5-HETE]}{Ki12})+[AA]}$$

$\frac{d[15-HPETE]}{dt}=\frac{K16[15-LOX][AA]}{k16(1+\frac{[15-HPETE]}{Ki})+[AA]}-\frac{K24[PHGPx][15-HPETE]}{k24(1+\frac{[15-HETE]}{Ki})+[15-HPETE]}$ $-Kd2*[15-HPETE]$

$$\frac{d[15-HETE]}{dt}=\frac{K24[PHGPx][15-HPETE]}{k24(1+\frac{[15-HETE]}{Ki})+[15-HPETE]}$$

$\frac{d[12-HPETE]}{dt}=\frac{K17[12-LOX][AA]}{k17(1+\frac{[12-HPETE]}{Ki18}+\frac{[15-HETE]}{Ki16})+[AA]}$ $-\frac{K24[PHGPx][12-HPETE]}{k24(1+\frac{[12-HETE]}{Ki})+[12-HPETE]}$

$$\frac{d[12-HETE]}{dt}=\frac{K24[PHGPx][12-HPETE]}{k24(1+\frac{[12-HETE]}{Ki})+[12-HPETE]}$$

$$\frac{d[PGH2]}{dt}=\frac{K18[COX][AA]}{k18(1+\frac{[PGH2]}{Ki}+\frac{[PGE2]}{Ki3})+[AA]}-\frac{K20[TXAS][PGH2]}{k20(1+\frac{[TXA2]}{Ki})+[PGH2]}$$

$$-\frac{K19[PGES][PGH2]}{k19(1+\frac{[PGE2]}{Ki}+\frac{[AA]}{Ki1}+\frac{[15-HETE]}{Ki2})+[PGH2]}$$

$$\frac{d[PGE2]}{dt}=\frac{K19[PGES][PGH2]}{k19(1+\frac{[PGE2]}{Ki}+\frac{[AA]}{Ki1}+\frac{[15-HETE]}{Ki2})+[PGH2]}$$

$$\frac{d[TXA2]}{dt}=\frac{K20[TXAS][PGH2]}{k20(1+\frac{[TXA2]}{Ki})+[PGH2]}-Kd8*[TXA2]$$

$$\frac{d[TXB2]}{dt}=Kd8*[TXA2]-Kd9*[TXB2]$$

$\frac{d[5-HPETE]}{dt}=\frac{K21[5-LOX][AA]}{k21(1+\frac{[5-HPETE]}{Ki}+\frac{[12-HETE]}{Ki7}+\frac{[15-HETE]}{Ki8}+\frac{[PGE2]}{Ki11}+\frac{[5-HETE]}{Ki12})+[AA]}$ $-\frac{K21[5-LOX][5-HPETE]}{k21(1+\frac{[LTA4]}{Ki}+\frac{[12-HETE]}{Ki7}+\frac{[15-HETE]}{Ki8}+\frac{[PGE2]}{Ki11}+\frac{[5-HETE]}{Ki12})+[5-HPETE]}$ $-\frac{K24[PHGPx][5-HPETE]}{k24(1+\frac{[5-HETE]}{Ki})+[5-HPETE]}$

$$\frac{d[5-HETE]}{dt}=\frac{K24[PHGPx][5-HPETE]}{k24(1+\frac{[5-HETE]}{Ki})+[5-HPETE]}-Kd11*[5-HETE]$$

$$\frac{d[LTA4]}{dt}=\frac{K21[5-LOX][5-HPETE]}{k21(1+\frac{[LTA4]}{Ki}+\frac{[12-HETE]}{Ki7}+\frac{[15-HETE]}{Ki8}+\frac{[PGE2]}{Ki11}+\frac{[5-HETE]}{Ki12})+[5-HPETE]}$$

$$-\frac{K22[LTA4H][LTA4]}{k22(1+\frac{[LTB4]}{Ki})+[LTA4]}-Kd12*[LTA4]$$

$\frac{d[LTB4]}{dt}=\frac{K22[LTA4H][LTA4]}{k22(1+\frac{[LTB4]}{Ki})+[LTA4]}-\frac{K23[CYP4F3][LTB4]}{k23(1+\frac{[20-OH-LTB4]}{Ki}+\frac{[12-HETE]}{Ki14}+\frac{[5-HETE]}{Ki15})+[LTB4]}$ $-Kd13*[LTB4]$

$$\frac{d[20-OH-LTB4]}{dt}=\frac{K23[CYP4F3][LTB4]}{k23(1+\frac{[20-OH-LTB4]}{Ki}+\frac{[12-HETE]}{Ki14}+\frac{[5-HETE]}{Ki15})+[LTB4]}$$

$$\frac{d[PLA2]}{dt}=0$$

$$\frac{d[15-LOX]}{dt}=\frac{a24*[PGE2]^{2}}{[PGE2]^{2}+KI24^{2}}$$

$$\frac{d[12-LOX]}{dt}=-Ki17*[15-HPETE][12-LOX]$$

$$\frac{d[COX]}{dt}=0$$

$$\frac{d[PGES]}{dt}=0$$

$$\frac{d[TXAS]}{dt}=-(Ki4*[15-HPETE]+Ki5*[PGH2])[TXAS]$$

$$\frac{d[5-LOX]}{dt}=(KI23*[LTB4]-Ki9*[LTA4]-Ki10*[5-HPETE]-Ki6*[15-HPETE])[5-LOX]$$

$$\frac{d[LTA4H]}{dt}=-\frac{K22[LTA4H][LTA4]}{129*(k22+[LTA4])}$$

$$\frac{d[CYP4F3]}{dt}=0$$

$$\frac{d[PHGPx]}{dt}=0$$

**Figure S1**. The arachidonic acid metabolic network in human PMNs. Metabolites (green), enzymes (blue) and feedback regulations (brown) are numbered for ordinary differential equations in SBML model.

**References**

1. Wijkander J, O'Flaherty JT, Nixon AB, Wykle RL (1995) 5-Lipoxygenase products modulate the activity of the 85-kDa phospholipase A2 in human neutrophils. J Biol Chem 270: 26543-26549.

2. Serio KJ, Baker JR, Ring WL, Riddick CA, Bigby TD (1997) Leukotriene B4 costimulates 5-lipoxygenase activity in neutrophils via increased 5-lipoxygenase translocation. Am J Physiol 272: C1329-1334.

3. Petrich K, Ludwig P, Kuhn H, Schewe T (1996) The suppression of 5-lipoxygenation of arachidonic acid in human polymorphonuclear leucocytes by the 15-lipoxygenase product (15S)-hydroxy-(5Z,8Z,11Z,13E)-eicosatetraenoic acid: structure-activity relationship and mechanism of action. Biochem J 314 ( Pt 3): 911-916.

4. Cashman JR, Lambert C, Sigal E (1988) Inhibition of human leukocyte 5-lipoxygenase by 15-HPETE and related eicosanoids. Biochem Biophys Res Commun 155: 38-44.

5. Lepley RA, Fitzpatrick FA (1994) Irreversible inactivation of 5-lipoxygenase by leukotriene A4. Characterization of product inactivation with purified enzyme and intact leukocytes. J Biol Chem 269: 2627-2631.

6. Orning L, Gierse J, Duffin K, Bild G, Krivi G, et al. (1992) Mechanism-based inactivation of leukotriene A4 hydrolase/aminopeptidase by leukotriene A4. Mass spectrometric and kinetic characterization. J Biol Chem 267: 22733-22739.

7. Aharony D, Redkar-Brown DG, Hubbs SJ, Stein RL (1987) Kinetic studies on the inactivation of 5-lipoxygenase by 5(S)-hydroperoxyeicosatetraenoic acid. Prostaglandins 33: 85-100.

8. Vanderhoek JY, Karmin MT, Ekborg SL (1985) Endogenous hydroxyeicosatetraenoic acids stimulate the human polymorphonuclear leukocyte 15-lipoxygenase pathway. J Biol Chem 260: 15482-15487.

9. Soberman RJ, Okita RT, Fitzsimmons B, Rokach J, Spur B, et al. (1987) Stereochemical requirements for substrate specificity of LTB4 20-hydroxylase. J Biol Chem 262: 12421-12427.

10. Levy BD, Clish CB, Schmidt B, Gronert K, Serhan CN (2001) Lipid mediator class switching during acute inflammation: signals in resolution. Nat Immunol 2: 612-619.

11. Kishimoto K, Nakamura M, Suzuki H, Yoshimoto T, Yamamoto S, et al. (1996) Suicide inactivation of porcine leukocyte 12-lipoxygenase associated with its incorporation of 15-hydroperoxy-5,8,11,13-eicosatetraenoic acid derivative. Biochim Biophys Acta 1300: 56-62.

12. Balboa MA, Perez R, Balsinde J (2003) Amplification mechanisms of inflammation: paracrine stimulation of arachidonic acid mobilization by secreted phospholipase A2 is regulated by cytosolic phospholipase A2-derived hydroperoxyeicosatetraenoic acid. J Immunol 171: 989-994.

13. Nathan C (2002) Points of control in inflammation. Nature 420: 846-852.

14. Jones DA, Fitzpatrick FA (1990) "Suicide" inactivation of thromboxane A2 synthase. Characteristics of mechanism-based inactivation with isolated enzyme and intact platelets. J Biol Chem 265: 20166-20171.

15. Jones DA, Fitzpatrick FA (1991) Thromboxane A2 synthase. Modification during "suicide" inactivation. J Biol Chem 266: 23510-23514.

16. Quraishi O, Mancini JA, Riendeau D (2002) Inhibition of inducible prostaglandin E(2) synthase by 15-deoxy-Delta(12,14)-prostaglandin J(2) and polyunsaturated fatty acids. Biochem Pharmacol 63: 1183-1189.

17. Powell WS, Gravelle F, Gravel S (1994) Phorbol myristate acetate stimulates the formation of 5-oxo-6,8,11,14-eicosatetraenoic acid by human neutrophils by activating NADPH oxidase. J Biol Chem 269: 25373-25380.

18. Serhan CN (1994) Lipoxin biosynthesis and its impact in inflammatory and vascular events. Biochim Biophys Acta 1212: 1-25.

19. Christmas P, Ursino SR, Fox JW, Soberman RJ (1999) Expression of the CYP4F3 gene. tissue-specific splicing and alternative promoters generate high and low K(m) forms of leukotriene B(4) omega-hydroxylase. J Biol Chem 274: 21191-21199.

20. Thoren S, Weinander R, Saha S, Jegerschold C, Pettersson PL, et al. (2003) Human microsomal prostaglandin E synthase-1: purification, functional characterization, and projection structure determination. J Biol Chem 278: 22199-22209.

21. Soberman RJ, Harper TW, Murphy RC, Austen KF (1985) Identification and functional characterization of leukotriene B4 20-hydroxylase of human polymorphonuclear leukocytes. Proc Natl Acad Sci USA 82: 2292-2295.

22. Schomburg I, Chang A, Ebeling C, Gremse M, Heldt C, et al. (2004) BRENDA, the enzyme database: updates and major new developments. Nucleic Acids Res 32: D431-433.
