# Supplementary material for: Dynamic Simulations on the Arachidonic Acid Metabolic Network
Source: PLoS Comput Biol. 2007 Mar 23;3(3):e55. doi: 10.1371/journal.pcbi.0030055 (PMC1829479; doi:10.1371/journal.pcbi.0030055)
Supplement: Supplementary file 2 [file pcbi.0030055.st001.doc]

**Table S1**. The feedback parameters used in ODE. Italics means there is no direct experimental value for the parameter and the value was derived by fitting calculated curves to the experimental data.

| Feedbacks | Parameters | Value |
| --- | --- | --- |
| AA→PGES[1] | Ki1 | 0.3μM |
| 15-HETE→PGES[1] | Ki2 | *30μM* |
| PGE2→COX[2] | Ki3 | *30μM* |
| 15-HPETE→TXA2S[3] | Ki4 | *0.6(μM*∙*min)^-1^* |
| PGH2→TXA2S[4] | Ki5 | *0.1(μM*∙*min)^-1^* |
| 15-HPETE→5-LOX[5] | Ki6 | 0.01(μM∙min)^-1^ |
| 12-HETE→5-LOX[6] | Ki7 | 30 μM |
| 15-HETE→5-LOX[7] | Ki8 | 4 μM |
| LTA4→5-LOX[8] | Ki9 | 0.175(μM∙min)^-1^ |
| 5-HPETE→5-LOX[9] | Ki10 | *0.01(μM∙min)^-1^* |
| PGE2→5-LOX[10] | Ki11 | *15μM* |
| 5-HETE→5-LOX[9] | Ki12 | 6.3μM |
| LTA4→LTA4H[11] | Ki13 | 129 (turnover/inactivation) |
| 12-HETE→CYP4F3[12] | Ki14 | *0.2μM* |
| 5-HETE→CYP4F3[12] | Ki15 | *0.86μM* |
| 15-HETE→12-LOX[13] | Ki16 | *10μM* |
| 15-HPETE→12-LOX[13] | Ki17 | *10(μM*∙*min)^-1^* |
| 12-HPETE→12-LOX[13] | Ki18 | *10μM* |
| 12-HPETE→PLA2[14] | KI19 | *500μM* |
| 15-HPETE→PLA2[14] | KI20 | *200μM* |
| LTB4→PLA2[15] | KI21 | *500μM* |
| 5-HETE→PLA2[15] | KI22 | *500μM* |
| LTB4→5-LOX[16] | KI23 | *0.053(μM*∙*min)^-1^* |
| PGE2→15-LOX[10] | a24  KI24 | 0.15  0.000023μM |

**References**

1. Quraishi O, Mancini JA, Riendeau D (2002) Inhibition of inducible prostaglandin E(2) synthase by 15-deoxy-Delta(12,14)-prostaglandin J(2) and polyunsaturated fatty acids. Biochem Pharmacol 63: 1183-1189.

2. Nathan C (2002) Points of control in inflammation. Nature 420: 846-852.

3. Jones DA, Fitzpatrick FA (1991) Thromboxane A2 synthase. Modification during "suicide" inactivation. J Biol Chem 266: 23510-23514.

4. Jones DA, Fitzpatrick FA (1990) "Suicide" inactivation of thromboxane A2 synthase. Characteristics of mechanism-based inactivation with isolated enzyme and intact platelets. J Biol Chem 265: 20166-20171.

5. Cashman JR, Lambert C, Sigal E (1988) Inhibition of human leukocyte 5-lipoxygenase by 15-HPETE and related eicosanoids. Biochem Biophys Res Commun 155: 38-44.

6. Vanderhoek JY, Karmin MT, Ekborg SL (1985) Endogenous hydroxyeicosatetraenoic acids stimulate the human polymorphonuclear leukocyte 15-lipoxygenase pathway. J Biol Chem 260: 15482-15487.

7. Petrich K, Ludwig P, Kuhn H, Schewe T (1996) The suppression of 5-lipoxygenation of arachidonic acid in human polymorphonuclear leucocytes by the 15-lipoxygenase product (15S)-hydroxy-(5Z,8Z,11Z,13E)-eicosatetraenoic acid: structure-activity relationship and mechanism of action. Biochem J 314 ( Pt 3): 911-916.

8. Lepley RA, Fitzpatrick FA (1994) Irreversible inactivation of 5-lipoxygenase by leukotriene A4. Characterization of product inactivation with purified enzyme and intact leukocytes. J Biol Chem 269: 2627-2631.

9. Aharony D, Redkar-Brown DG, Hubbs SJ, Stein RL (1987) Kinetic studies on the inactivation of 5-lipoxygenase by 5(S)-hydroperoxyeicosatetraenoic acid. Prostaglandins 33: 85-100.

10. Levy BD, Clish CB, Schmidt B, Gronert K, Serhan CN (2001) Lipid mediator class switching during acute inflammation: signals in resolution. Nat Immunol 2: 612-619.

11. Orning L, Gierse J, Duffin K, Bild G, Krivi G, et al. (1992) Mechanism-based inactivation of leukotriene A4 hydrolase/aminopeptidase by leukotriene A4. Mass spectrometric and kinetic characterization. J Biol Chem 267: 22733-22739.

12. Soberman RJ, Okita RT, Fitzsimmons B, Rokach J, Spur B, et al. (1987) Stereochemical requirements for substrate specificity of LTB4 20-hydroxylase. J Biol Chem 262: 12421-12427.

13. Kishimoto K, Nakamura M, Suzuki H, Yoshimoto T, Yamamoto S, et al. (1996) Suicide inactivation of porcine leukocyte 12-lipoxygenase associated with its incorporation of 15-hydroperoxy-5,8,11,13-eicosatetraenoic acid derivative. Biochim Biophys Acta 1300: 56-62.

14. Balboa MA, Perez R, Balsinde J (2003) Amplification mechanisms of inflammation: paracrine stimulation of arachidonic acid mobilization by secreted phospholipase A2 is regulated by cytosolic phospholipase A2-derived hydroperoxyeicosatetraenoic acid. J Immunol 171: 989-994.

15. Wijkander J, O'Flaherty JT, Nixon AB, Wykle RL (1995) 5-Lipoxygenase products modulate the activity of the 85-kDa phospholipase A2 in human neutrophils. J Biol Chem 270: 26543-26549.

16. Serio KJ, Baker JR, Ring WL, Riddick CA, Bigby TD (1997) Leukotriene B4 costimulates 5-lipoxygenase activity in neutrophils via increased 5-lipoxygenase translocation. Am J Physiol 272: C1329-1334.
