# Supplementary material for: Dynamic Simulations on the Arachidonic Acid Metabolic Network
Source: PLoS Comput Biol. 2007 Mar 23;3(3):e55. doi: 10.1371/journal.pcbi.0030055 (PMC1829479; doi:10.1371/journal.pcbi.0030055)
Supplement: Supplementary file 3 [file pcbi.0030055.st002.doc]

**Table S2**. The K_m_ and K_cat_ of enzymes used in the model. Italics means no direct value for this parameter can be found in literature and its value was derived by fitting calculated curves to the experimental data.

| Enzyme Name | EC number | K_m_（μM） | K_cat_（1/min） |
| --- | --- | --- | --- |
| 15-LOX | 1.13.11.33[1] | *70* | *1000* |
| 5-LOX | 1.13.11.34[1] | *5* | *5000* |
| LTA4H | 3.3.2.6[1] | 20 | 125 |
| CYP4F3 | 1.14.13.30[2] | 3.9 | *150* |
| PHGPx | 1.11.1.12[1] | *70* | *500* |
| COX-2 | 1.14.99.1[1] | *50* | 1000 |
| PGES | 5.3.99.3[1] | 160 | 3000 |
| TXAS | 5.3.99.5[1] | 4 | 1599 |
| PLA2 | 3.1.1.4[1] | 2600 | 3600 |
| 12-LOX | 1.13.11.31[1] | *50* | *1000* |

**References**

1. Schomburg I, Chang A, Ebeling C, Gremse M, Heldt C, et al. (2004) BRENDA, the enzyme database: updates and major new developments. Nucleic Acids Res 32: D431-433.

2. Christmas P, Ursino SR, Fox JW, Soberman RJ (1999) Expression of the CYP4F3 gene. tissue-specific splicing and alternative promoters generate high and low K(m) forms of leukotriene B(4) omega-hydroxylase. J Biol Chem 274: 21191-21199.
